# Repeated measures of physical activity before dementia diagnosis in community-dwelling older adults: a longitudinal study

**DOI:** 10.1016/j.lanhl.2026.100824

**Published:** 2026-02

**Authors:** Shahram Oveisgharan, Jingyun Yang, Tianhao Wang, David A Bennett, Aron S Buchman

**Affiliations:** Rush Alzheimer’s Disease Center, Rush University Medical Center, Chicago, IL, USA (S Oveisgharan MD, J Yang PhD, T Wang PhD, D A Bennett MD, A S Buchman MD); Department of Neurological Sciences, Rush University Medical Center, Chicago, IL, USA (S Oveisgharan, J Yang, T Wang, D A Bennett, A S Buchman)

## Abstract

**Background:**

Many studies have reported that a single measure of physical activity in older adults is associated with incident dementia. In this study, we examined repeated measures of physical activity to determine if this association with incident dementia varied.

**Methods:**

Participants were from a community-based longitudinal study of older adults (the Rush Memory and Aging Project [MAP]). MAP participants were recruited from retirement centres and subsidised long-term care facilities across northeastern Illinois (USA). Physical activity level was extracted from biennial multiday wrist-wearing sensor recordings. Dementia diagnosis was based on neuropsychological test scores, clinical history, and examination. Joint modelling integrating a linear mixed-effects model and a Cox model was used to prospectively test the association of repeated measures of physical activity and dementia, and a time-varying effects model was used to retrospectively examine the association of dementia with repeated measures of physical activity before dementia diagnosis.

**Findings:**

Our analyses included 972 older adults (mean age 80·5 years, SD 7·3) with frequent (mean 4·9, SD 2·6) biennial measurements of physical activity. 745 (77%) of 972 participants were female and 227 (23%) were male. 286 (29%) of the 972 participants developed dementia, which was clinically diagnosed as Alzheimer’s disease. Although the joint model indicated an association between physical activity and incident dementia (hazard ratio [HR] 0·78, 95% CI 0·69–0·88; p<0·0001), the model accuracy was stronger for physical activity closer to dementia diagnosis. Baseline physical activity was not related to the risk of dementia more than 7 years after baseline (HR 1·00, 95% CI 0·69–1·45; p=0·99), whereas physical activity at year 6 was (0·55, 0·37–0·80; p=0·0021). In a time-varying effects model, repeated physical activity was not associated with incident dementia except during the last 2 years before dementia diagnosis.

**Interpretation:**

Physical activity might be a modifiable protective factor for dementia in old age. However, analyses of repeated measures of physical activity suggest that further work is needed to define the timing of the beneficial effects of physical activity relative to the onset of dementia.

**Funding:**

National Institutes of Health.

## Introduction

In the absence of effective drugs that prevent dementia, a physically active lifestyle is commonly recommended for prevention in older adults without cognitive impairment^1^ and adults with mild cognitive impairment (MCI).^2^ The 2024 *Lancet* dementia Commission suggested that physical activity might prevent or delay dementia through multiple pathways, such as reducing dementia neuropathology, reducing stress and inflammation, or building cognitive and brain reserve.^1^ These recommendations for physical activity are based on longitudinal observational studies that reported an association between a single measure of increased physical activity and a reduced risk of dementia.^3^

Research suggests that accumulation of dementia-related pathologies, such as those of Alzheimer’s disease, might occur many years before clinical evidence of cognitive impairment or dementia. Moreover, the negative effects of accumulating dementia-related pathologies are not limited to cognition; non-cognitive phenotypes such as gait and mobility might also be affected.^4,5^ Hence, the observed association between physical activity and incident dementia in older adults might not represent a causal association between increased physical activity and reduced risk of dementia. Rather, this association might be a consequence of reverse causality due to accumulation of dementia-related pathologies during the pre-dementia stages,^6^ which might degrade motor abilities^4^ or cause apathy,^7^ resulting in reduced engagement in physical activity.^8^ The average follow-up time between baseline physical activity and incident dementia in previous studies of older adults was 4–6 years, which is much lower than the estimated 10-year duration of the pre-dementia stages of Alzheimer’s disease.^9^

Many studies analysing repeated measures of physical activity in older adults have used linear models, such as linear mixed-effects models. These models are based on assumptions that repeated measures of physical activity change linearly over time and are uniformly associated with the outcome over time. Recent work has suggested that both linear and non-linear modelling might be necessary to capture the complexity of declining cognitive and motor function in ageing adults.^10^

To account for gaps in previous studies, we examined the association between repeated measures of physical activity and incident dementia and extended our previous work^11^ that focused on the association between a single baseline level of physical activity and risk of dementia. We used multiple measures of physical activity to examine the time course of the association between physical activity and dementia using linear and non-linear models and to test reverse causality between physical activity and dementia. We also used Mendelian randomisation to investigate the association between physical activity and Alzheimer’s disease using publicly available datasets.

## Methods

### Participants

Participants were from the Rush Memory and Aging Project (MAP), an ongoing community-based longitudinal study of ageing. MAP participants were recruited from the community, including retirement centres and subsidised long-term care facilities across northeastern Illinois (USA). From 1997 until the present day, 54 sites have been participating in MAP, but the current participants were from 24 sites. The participants were without known dementia at enrolment and consented to annual clinical evaluations. Ethics approval of MAP was given by an Institutional Review Board at Rush University Medical Center. All participants provided written informed consent.

Although MAP enrolment began in September, 1997, deployment of sensors for quantifying physical activity began in 2005. Therefore, the analytical baseline for the current study was the first cycle when the sensor-derived level of daily physical activity was measured, and follow-up visits were included until the first diagnosis of dementia, death, or the last follow-up visit, whichever occurred first. Other inclusion criteria were no dementia at analytical baseline and having at least one valid follow-up measurement of physical activity. Sex and race and ethnicity were self-reported by participants.

### Sensor-derived physical activity measure

Sensors were used to measure physical activity to circumvent the limitations of self-reporting^12^ and recall bias in older adults with possible cognitive impairment. Participants were instructed to wear a wrist-sensor on their non-dominant wrist continuously for 10 days (Actical; Philips Healthcare, Bend, OR, USA). The devices were placed and removed by a research assistant between 0900 h and 1700 h (Monday to Friday). To facilitate alignment of data between participants, we excluded data from before 1900 h on the day of placement and incomplete recordings on the days the devices were retrieved, which allowed us to analyse only complete days of 24-h recordings. We summed activity counts of 15-s epochs during each 24 h of recording. Daily sums of activity counts were averaged across the 10 days to yield a mean total daily physical activity count. Previous studies have reported activity counts measured during various physical activities and performances in older adults. For example, walking at 2·5 mph is associated with 2354 activity counts per min.^13^ Further detail on the sensor-derived physical activity measure is provided elsewhere.^8^ As was done previously, sensor-derived physical activity was log-transformed for longitudinal modelling.^8^

The intensity of physical activity might affect the potential health benefits. An indirect measure of the intensity of physical activity was developed by dividing the total daily activity count by total h per day of non-zero epochs, which results in activity count per h, with higher numbers indicative of more activity counts per h and more vigorous physical activity.^14^ The activity count per h was also log-transformed.

### Dementia diagnosis

21 neuropsychological tests were administered to participants once per year, and scores were standardised and averaged to make a global cognition score.^10^ Participant scores were reviewed by a neuropsychologist to determine cognitive impairment. An expert clinician reviewed clinical data, including the neuropsychologist’s ratings and the neurological examinations, and adjudicated the presence of dementia, MCI, or no cognitive impairment.^15^ Only clinical data, including neuropsychological test ratings, were used to make the dementia diagnosis and identify any possible underlying causes because study enrolment started in 1997, when Alzheimer’s disease biomarkers were not available.

### Other covariates

Other covariates were age, sex, race, ethnicity, education, vascular risk factors and diseases (including BMI), depressive symptoms, having purpose in life, participation in cognitive and social activities, presence of apolipoprotein E *(APOE)* ε4 allele, parkinsonism, and parkinsonism severity. Further information about the covariates is provided in the appendix (p 4).

### Statistical analysis

For bivariate comparison of participants who did or did not develop dementia, *t* test (continuous variables), χ^2^ test (categorical variables), and Kruskal-Wallis (ordinal variables) tests were used. We analysed longitudinal physical activity measures and incident dementia with a shared-parameter joint model. In this framework, each participant’s activity trajectory is described by a linear mixed-effects submodel with random intercepts and slopes, while a proportional hazards submodel relates the continuously updated, person-specific level of physical activity to dementia risk.^16^ The joint modelling was implemented using the JMbayes package in R version 4.4.1.^17^ More information about the joint modelling is provided in the appendix (p 5). To assess the accuracy of the joint model in predicting participants who developed dementia, we calculated the area under the curve (AUC) of the time-dependent receiver operating characteristic curves derived from the joint models examining 1 to 6 years of physical activity measurements in relation to the risk of dementia during the next 7 years of follow-up. For comparison, we examined time-invariant Cox models to observe the associations between baseline physical activity and risk of dementia or parkinsonism at different timepoints or the associations between a single measurement of physical activity at different timepoints and incident dementia 7 years or more after baseline.

In the joint model, a constant association was assumed between physical activity and dementia. To test if the association between physical activity and incident dementia varied over several years of physical activity measurements, we used a time-varying effects model, as this model imposes minimum assumptions on the trajectory of change in physical activity and allows a non-linear time-varying association between physical activity and incident dementia.^18^ For the time-varying effects model analysis, we created matched incident dementia and control cases, as participants with and without incident dementia generally differed in age or duration of follow-up. We created the matched case and control groups by matching those with incident dementia (n=285) with those without incident dementia (n=285) on duration of follow-up, age at the time of incident dementia (±3 years), and sex. The cases and controls were aligned at the time of incident dementia (which was defined as time zero).

To test for reverse causality—ie, that low physical activity during pre-dementia is a consequence of Alzheimer’s disease and other causes of dementia—we used linear mixed-effects models and examined the association between baseline MCI and global cognition and changes in physical activity over time. The models controlled for age at baseline, sex, education, and interactions with time. We also used a time-varying effects model that compared participants with and without MCI at baseline, matched by age at baseline (±3 years), sex, and duration of follow-up (±3 years). The latter model evaluated a possible non-linear association between lower cognition at baseline (MCI status *vs* no cognitive impairment) with repeated measures of the level of physical activity during follow-up. All models described above were adjusted for age, sex, and education.

To examine the causal relationship between physical activity and Alzheimer’s disease risk, we did a two-sample Mendelian randomisation analysis, with a multiplicative random effects inverse-variance weighted model as the main analytical tool. We also used other models for sensitivity analyses, including Egger and weighted median methods, to assess the robustness of the findings and detect potential pleiotropy. Further details about the Mendelian randomisation analyses, including instrumental variable selection, are provided in the appendix (pp 4–5).

### Role of the funding source

The funder of the study had no role in study design, data collection, data analysis, data interpretation, or writing of the report.

## Results

Of 1398 MAP participants with completed baseline evaluation, 197 did not have valid sensor-derived physical activity and 229 did not have a valid follow-up assessment, including physical activity recording, leaving 972 adults analysed in the current study. 745 (77%) of 972 participants were female and 227 (23%) were male. The mean age was 80·5 years (SD 7·3). Comparison of these three groups of MAP participants (972 included in the current study, 197 without physical activity assessment, and 229 without a valid follow-up) indicated that the groups did not differ in terms of sex, race and ethnicity, and vascular risk factors, but were different in terms of age, engagement in cognitive and social activities, having purpose in life, and history of dementia or stroke (appendix p 3).

286 (29%) of 972 people developed dementia. The median baseline daily activity count in the 286 people with incident dementia was 2·59 × 10^5^ (IQR 1·62–3·72), equivalent to 110·0 min (68·8–158·0) of walking at 2·5 mph (table 1).^13^ Median follow-up was 7·0 years (IQR 5·0–10·0; range 1·0–17·0) with about five repeated measures of physical activity per participant (mean 4·9, SD 2·6; range 2–13). Participants with incident dementia were older at baseline, were more likely to carry the *APOE ε4* allele, and more likely to have MCI at baseline than patients without incident dementia (table 1).

The linear mixed-effects submodel of the joint model indicated that, on average, physical activity declined over time in older adults, as reflected by a negative estimate of the slope term (estimate −0·1465, SE 0·0001; p<0·0001), corresponding to an annual decline of 13·6% in the level of physical activity. The proportional hazards submodel revealed that increased levels of physical activity across the follow-up period were associated with a reduced risk of dementia (hazard ratio [HR] 0·78, 95% CI 0·69–0·88; p<0·0001). This association remained robust after controlling for baseline vascular risk factors, BMI, stroke history, depressive symptoms, having purpose in life, cognitive and social activity, MCI, *APOE ε4*, and parkinsonism severity score (table 2; appendix pp 7, 9–10).

**Figure 1:**
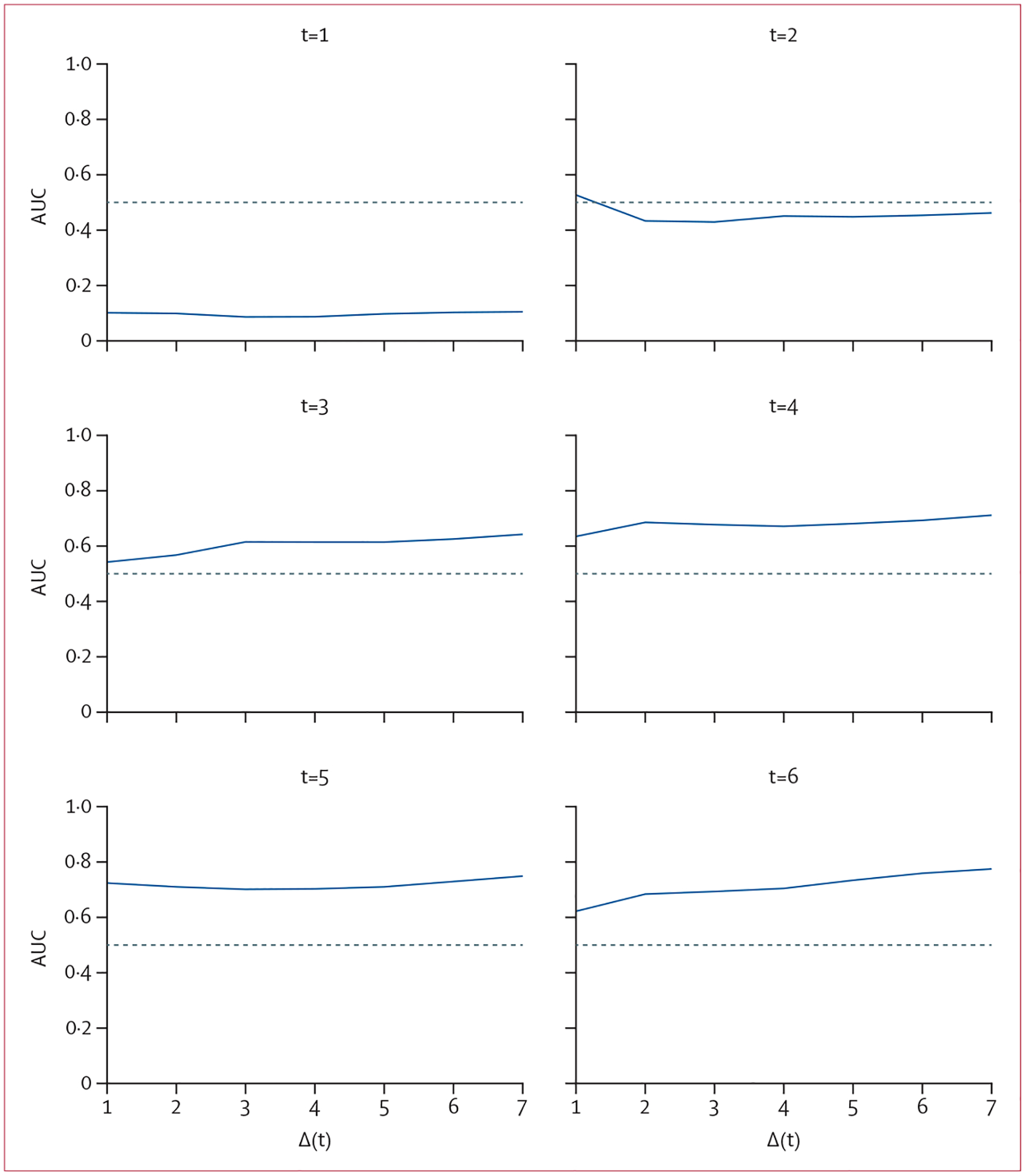
AUC of risk prediction for dementia by physical activity level at different years of follow-up Each panel shows the AUC of risk prediction for dementia by level of physical activity derived from a joint model examining 1 (t=1) to 6 (t=6) years of physical activity measurements in relation to the risk of dementia during the next 7 years. The x-axis indicates the year of follow-up. AUC=area under the curve.

Based on the observed association between repeated measures of physical activity and dementia risk, we did supplementary analyses to assess the accuracy of the joint model in identifying those who would develop dementia. The median onset of incident dementia was 6·0 years (IQR 4·0–10·0; range 1·0–17·0) from baseline. Using AUC as the measure of accuracy, we found that models incorporating physical activity data close to the onset of dementia were more accurate. For example, a model using up to 3 years of physical activity data yielded an AUC of 0·61 in correctly identifying those developing dementia within the following 5 years. By contrast, a model using 5 years of physical activity data achieved an AUC of 0·71 in correctly identifying those developing dementia within the next 2 or 5 years (figure 1).

**Figure 2:**
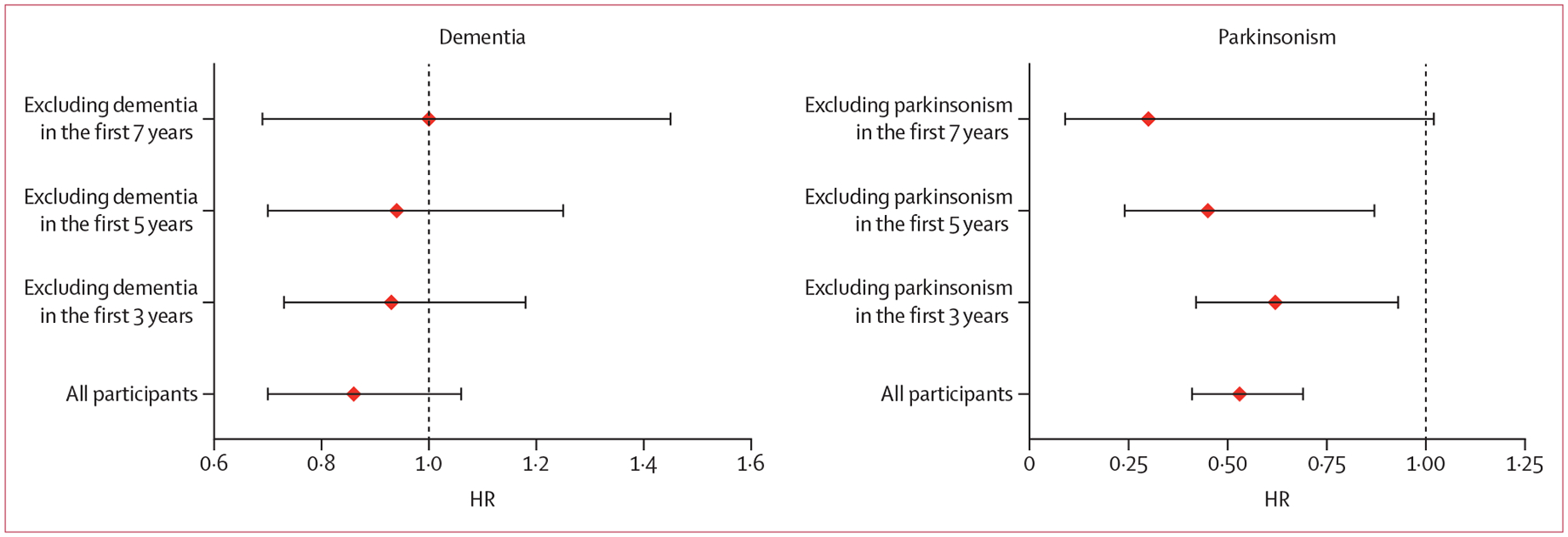
HR of risk of dementia or parkinsonism by baseline level of physical activity after excluding early dementia or parkinsonism cases Data show the HRs (red diamonds) and 95% CIs (error bars) of the association of baseline physical activity with the risk of dementia (left panel) or parkinsonism (right panel). HR=hazard ratio.

To corroborate these findings, we did two sets of complementary Cox regression analyses. First, we used physical activity level only at baseline and progressively excluded participants who developed dementia within the first 3, 5, or 7 years of follow-up. This approach lengthened the interval between physical activity assessment and dementia diagnosis. As expected, the strength of the association diminished when increasing the interval (figure 2). The HR for baseline physical activity and dementia risk was 0·86 (95% CI 0·70–1·06; p=0·15) when no cases were excluded (286 dementia cases), and attenuated to 0·93 (0·73–1·18; p=0·55), 0·94 (0·70–1·25; p=0·66), and 1·00 (0·69–1·45; p=0·99) after excluding dementia cases occurring within the first 3, 5, or 7 years (number of dementia cases 236, 172, and 122, respectively).

For comparison, we applied the same exclusion approach to a previously reported association between physical activity and incident parkinsonism^14^ in a subset of participants with available parkinsonism measurements and without parkinsonism at baseline (n=611). Unlike dementia, the protective association between baseline physical activity and the risk of parkinsonism remained strong even when early cases were excluded, with HRs of 0·53 (all cases of parkinsonism, n=208), 0·62 (including only cases of parkinsonism occurring ≥3 years from baseline), 0·45 (≥5 years), and 0·30 (≥7 years; figure 2).

In the second set of Cox models, we restricted dementia cases to those occurring at least 7 years after baseline and examined the association between physical activity levels at different timepoints (baseline through to year 6) and subsequent dementia risk, increasing the interval between exposure and outcome. These models consistently showed that physical activity levels measured closer to dementia onset were more strongly associated with reduced dementia risk. Although baseline physical activity was not associated with dementia (HR 1·00), physical activity levels at years 2, 4, and 6 were increasingly associated with reduced dementia risk (HR 0·61, 0·43–0·86; p=0·0050; 0.61, 0·44–0·85; p=0·0032; and 0.55, 0·37–0·80; p=0·0021, respectively; appendix p 11). These complementary findings supported the joint modelling findings that recent physical activity is a strong correlate of future dementia risk.

**Figure 3:**
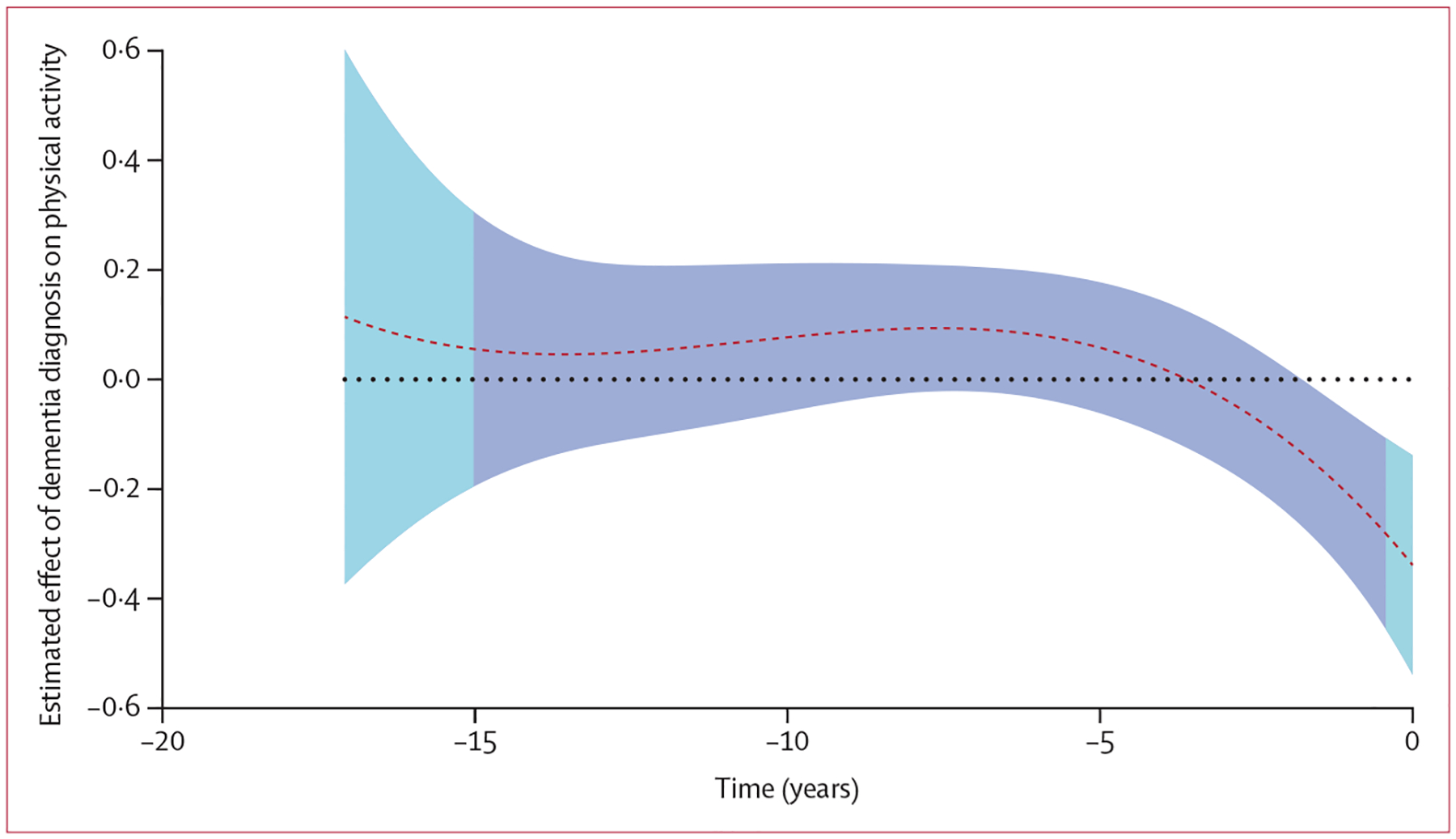
Retrospective association between dementia and repeated measures of physical activity before dementia diagnosis Dark blue shading indicates the area with the densest physical activity data, and light blue shading indicates where physical activity data are more sparse. The red dotted line is the point estimate of the association between dementia and physical activity while the black dotted line is the line of zero indicating no difference between participants with and without dementia in physical activity. When the red dotted line and its confidence interval (the shaded areas) cover the black dotted line of zero, there is no association between dementia and physical activity.

The above analyses suggested that the association between physical activity and incident dementia might vary over time. To statistically model the temporal association between repeated measures of physical activity and dementia, we used a time-varying effects model. The time-varying effects model showed that, on average, physical activity was declining in all participants (appendix p 12), but the level of physical activity did not differ between those who did or did not develop dementia until 1·8 years before dementia diagnosis, when activity was lower in those subsequently diagnosed with dementia (figure 3). These findings did not change when we adjusted the models for vascular risk factors, stroke, depressive symptoms, purpose in life, cognitive and social activity, MCI, and *APOE ε4* (appendix p 13).

Association between low levels of physical activity during the years preceding dementia diagnosis and dementia could occur because low physical activity is a risk factor for dementia or could be reverse causality (ie, because accumulating dementia-related pathologies cause low physical activity in the pre-dementia stage). To test for reverse causality, we examined the association between baseline MCI status or a low global cognition score, as markers of Alzheimer’s disease and other dementia-related pathologies, with longitudinal changes in physical activity. In two separate linear mixed-effects models, baseline MCI status and baseline global cognition score were not associated with the rate of declining physical activity (appendix pp 14–15). Baseline MCI was also not associated with longitudinal levels of physical activity in a time-varying effects model (appendix p 16).

To further investigate the relationship between physical activity and dementia, we did a two-sample Mendelian randomisation analysis to assess a potential causal effect of physical activity on Alzheimer’s disease dementia risk. 16 genetic variants associated with physical activity in individuals of European ancestry^19^ were used as instrumental variables (appendix p 17). The genetic instruments showed adequate strength, with F statistics for all variants exceeding ten (range 30–81). The Mendelian randomisation analysis revealed no association between genetically predicted levels of physical activity and Alzheimer’s disease dementia risk (appendix p 18). To evaluate potential reverse causality, we did an additional Mendelian randomisation analysis using 58 genetic variants associated with Alzheimer’s disease dementia susceptibility in European ancestry^20^ (appendix pp 19–23) as instruments to test whether genetic liability to Alzheimer’s disease dementia affected physical activity levels. The genetic instruments showed adequate strength, with F statistics for all variants exceeding ten (range 30–519). This analysis similarly showed no evidence of an effect of Alzheimer’s disease genetic risk on physical activity (appendix p 24).

## Discussion

In this study of older adults with repeated measures of daily physical activity, we found that reduced levels of physical activity, when measured close to the time of dementia, were strongly associated with dementia risk. In a matched analysis, a significant difference in physical activity between people with dementia and those without dementia emerged in the last 2 years before dementia diagnosis. Baseline MCI status and global cognition score were not associated with declining physical activity, suggesting that the association between physical activity and dementia is not a consequence of accumulating dementia-related pathologies in the pre-dementia stage. These findings suggest that higher levels of physical activity are associated with a lower dementia risk primarily in the years preceding the clinical onset of dementia. These results also highlight the need for clinical trials to assess the optimal timing and duration of increased physical activity intervention to delay the onset of dementia in older adults.

The absence of clinically effective disease-modifying therapies for dementia has shifted attention towards modifiable lifestyle factors such as physical activity, which might prevent or slow down the dementia process. Findings from many observational studies^3^ support recommendations to older adults to engage in physical activity to prevent or delay the risk of dementia.^1,21^ However, most of these studies relied on a single measure of physical activity and could not disentangle causality and reverse causation. Alzheimer’s disease and related pathologies are chronic diseases in which brain pathologies accumulate for years before clinical manifestations of dementia.^6^ Consequently, when an observational study finds a low level of physical activity at baseline as a risk factor for incident dementia, it is uncertain whether a low level of physical activity is a risk factor for dementia, or whether the pre-dementia stage of the accumulating pathologies leads to low physical activity. In the latter situation, low physical activity is a marker or a manifestation of the dementia process, but not a risk factor. By analysing repeated, objective measures of daily physical activity, our study extended previous studies and found that the repeated measures of physical activity were associated with incident dementia, and the association was unlikely to be a consequence of Alzheimer’s disease and related pathologies as the presence of MCI and low global cognition score at baseline was not associated with the rate of physical activity decline during follow-up.

Resilience refers to the capacity of the brain to maintain function despite accumulating pathologies and ageing-related changes.^22^ The negative association between reduced levels of physical activity during the final years before diagnosis and dementia might reflect a role of physical activity in promoting resilience. During the final years before dementia diagnosis, Alzheimer’s disease and related pathologies are already present in the brain but have not reached a threshold^22^ of damage for the manifestation of dementia. High levels of physical activity might elevate the threshold of dementia manifestation by providing brain reserve, such as building new synapses through activation of pathways, including brain-derived neurotrophic factor and irisin.^23^ This hypothesis is supported by observational studies^24^ and randomised clinical trials examining physical activity in patients with MCI.^25^

Our findings could have important implications for public health policies if confirmed by future intervention studies. Physical activity is widely recommended by experts and health organisations as a lifestyle intervention to prevent dementia in the community.^1,21^ However, our findings suggest that for cognitively unimpaired older adults who are not at an imminent risk of dementia, physical activity might not prevent dementia that develops years or decades later, suggesting that other preventive measures need to be developed. This finding aligns with an umbrella review of randomised clinical trials, which reported low, if any, benefit of physical activity on cognitively healthy individuals.^26^

Our Mendelian randomisation analysis did not show any association between physical activity and Alzheimer’s disease dementia, which could indicate no causal association between physical activity and dementia, or could be a consequence of certain limitations, such as timing mismatch. Physical activity is a time-varying exposure variable; however, previous summary statistics used to portray the association between genetic variants and physical activity used a single timepoint assessment of physical activity, whereas in the current study we showed that repeated measures of physical activity are associated with the risk of dementia primarily during the last years before dementia diagnosis. Therefore, further genetic association studies, including multiple assessments of physical activity over the lifetime, are required to derive the trajectory of physical activity over time and to implement Mendelian randomisation analysis using the trajectory of physical activity.^27^ Such complex Mendelian randomisation analyses might support the conclusion that no association between physical activity and dementia in the Mendelian randomisation of this study is because of timing mismatch. Nevertheless, the Mendelian randomisation analysis finding that Alzheimer’s disease dementia was not associated with physical activity supports our finding that the baseline level of cognition and MCI were not associated with the slope of physical activity decline.

Several limitations of our study must be noted. Despite the longitudinal design, this study is observational and did not consider biomarkers of Alzheimer’s disease or other dementia-related pathologies, therefore it is unable to reliably separate Alzheimer’s disease and non-Alzheimer’s disease causes of dementia. Further clinical trials are needed to allow for causal inferences with respect to the associations reported in this study. The analytical sample included primarily highly educated White volunteers in their ninth decade of life, and our study findings might not be generalisable to older adults at younger ages or from other socioeconomic or ethnic groups. Nevertheless, our study has notable strengths, including a large, well characterised cohort of older adults with objectively measured physical activity over an average of 5 years, and the use of advanced statistical techniques to examine the association between longitudinal measurements of physical activity and dementia risk.

In conclusion, our findings support an association between physical activity and reduced dementia risk, and extend this association to a specific time window close to dementia diagnosis. By using repeated measurements of physical activity, we tested the direction of association between physical activity and dementia—low physical activity is a risk factor for dementia, but it is unlikely that dementia-related brain pathologies, including Alzheimer’s disease, are causing low physical activity in the pre-dementia stage. Our findings imply that modifiable factors other than physical activity should be sought for prevention of dementia in older adults who are not at an imminent risk of dementia, and confirm that physical activity for prevention of dementia in high-risk older people requires further investigation and consideration of the timing of intervention relative to dementia risk.

## Supplementary Material

1

## Figures and Tables

**Table 1:** Baseline characteristics

	Incident dementia (n=286)	No incident dementia (n=686)
Age at baseline, years	83·4 (6·0)	79·3 (7·4)
Sex		
Female	224 (78%)	521 (76%)
Male	62 (22%)	165 (24%)
Race and ethnicity		
Latino	5 (2%)	22 (3%)
Non-Latino White	267 (93%)	625 (91%)
Non-Latino Black	14 (5%)	34 (5%)
Other*	0	5 (1%)
Years of education	15·1 (3·1)	15·2 (2·9)
**Clinical characteristics**		
Hypertension	162 (57%)	399 (58%)
Diabetes	34 (12%)	99 (14%)
Any history of smoking	103 (36%)	289 (42%)
Number of vascular risk factors	1·0 (0·0–2·0)	1·0 (1·0–2·0)
BMI, kg/m^2^	26·8 (5·2)	27·6 (5·5)
History of stroke	26/260 (10%)	65/619 (11%)
Apolipoprotein E ε4 allele	87/281 (31%)	113/669 (17%)
Number of depressive symptoms	0·0 (0·0–1·0)	0·0 (0·0–1·0)
Purpose in life	3·7 (3·4–3·9)	3·8 (3·5–4·0)
Social activity	2·67 (2·33–3·00)	2·67 (2·33–3·17)
Cognitive activity	3·21 (2·86–3·57)	3·29 (2·86–3·71)
Mild cognitive impairment	97 (34%)	92 (13%)
Daily activity count	2·59 × 10^5^ (1·62–3·72)	2·47 × 10^5^ (1·66–3·55)
Years from baseline to dementia or end of follow-up	6·0 (4·0–10·0)	8·0 (5·0–10·0)

Data are mean (SD), n (%), median (IQR), or n/N (%). For comparing characteristics across the two groups, *t*-test (continuous variables), Chi-square (categorical variables), and Kruskal-Wallis (ordinal variables) tests were used. Purpose in life was measured by Ryff’s scales of Psychological Well-Being, cognitive activity by asking seven questions about participation in cognitive activities, such as reading books, and social activity by asking six questions about participation in social activities, such as going to restaurants and visiting friends and families.

* Races and ethnicities with a frequency less than five are included under other.

**Table 2:** Association of repeated measures of physical activity with the risk of dementia

	Hazard ratio (95% CI); p-value
Longitudinal level of physical activity (reference model)	0·78 (0·69–0·88); <0·0001
Longitudinal level of physical activity plus summary score of vascular risk factors plus BMI	0·79 (0·69–0·89); <0·0001
Longitudinal level of physical activity plus summary score of vascular risk factors plus BMI plus stroke	0·78 (0·67–0·91); <0·0001
Longitudinal level of physical activity plus summary score of vascular risk factors plus BMI plus stroke plus depressive symptoms plus having purpose in life	0·79 (0·69–0·90); <0·0001
Longitudinal level of physical activity plus summary score of vascular risk factors plus BMI plus stroke plus depressive symptoms plus having purpose in life plus social activity plus cognitive activity	0·80 (0·68–0·93); 0·0061
Longitudinal level of physical activity plus summary score of vascular risk factors plus BMI plus stroke plus depressive symptoms plus having purpose in life plus social activity plus cognitive activity plus MCI	0·80 (0·69–0·92); 0·0033
Longitudinal level of physical activity plus summary score of vascular risk factors plus BMI plus stroke plus depressive symptoms plus having purpose in life plus social activity plus cognitive activity plus MCI plus *APOE ε4*	0·76 (0·65–0·89); <0·0001
Longitudinal level of physical activity plus summary score of vascular risk factors plus BMI plus stroke plus depressive symptoms plus having purpose i n life plus social activity plus cognitive activity plus MCI plus *APOE ε4* plus parkinsonism severity score	0·79 (0·67–0·92); 0·0033

In eight separate joint models, the association between the repeated measures of physical activity and the risk of dementia was examined. The model terms are included in the second column. Additionally, all the models were controlled for age at baseline, sex, and education. *APOE ε4*=apolipoprotein E. MCI=mild cognitive impairment.

## Data Availability

The data are available via the Rush Alzheimer’s Disease Center Research Resource Sharing Hub at www.radc.rush.edu. Qualified applicants should fill in an application including study premises and a brief description of their research plan.

## Research in context

### Evidence before this study

To review the literature related to the association between physical activity and dementia and Alzheimer’s disease in older adults, we searched PubMed between database inception and Aug 18, 2025, with the following terms: “physical activity” AND (“Alzheimer’s disease” OR “dementia”) AND (older OR elder) AND (longitudinal OR cohort), with no language restrictions. The query retrieved 566 articles. Most articles examined the baseline level of physical activity, either objectively or subjectively measured, in relation to the risk of cognitive impairment and dementia. Generally, these articles reported an inverse association between physical activity and dementia—higher physical activity was associated with a lower risk of dementia or vice versa. However, few studies examined repeated measures of physical activity to assess if they were associated with incident dementia and whether the associations varied over time.

### Added value of this study

In the current study, we examined frequent measurements of physical activity in relation to the risk of dementia, clinically diagnosed as Alzheimer’s disease dementia, in 286 of 972 included participants who went on to be diagnosed with Alzheimer’s disease. Using a joined mixed-effects model and Cox model, we found that multiple measurements of physical activity were associated with incident dementia. However, a model accuracy calculation revealed that incorporating physical activity data close to the onset of dementia provided more accurate results. Complementary Cox model analyses showed that the baseline physical activity was not associated with the risk of dementia more than 7 years after baseline, whereas physical activity at year 6 was. In a time-varying effects model in a subset of 285 matched dementia cases and controls, matched by age, sex, and duration of follow up, repeated physical activity was not related to incident dementia, except during the last 2 years before dementia diagnosis. Therefore, the current study adds to previous evidence by supporting the association between physical activity and dementia and suggesting a time window, the years close to dementia diagnosis, where the association between physical activity and dementia is strongest. The study also shows that reverse causality—ie, dementia-related pathologies including Alzheimer’s disease cause low physical activity—is unlikely to underlie the association between physical activity and dementia, as cognition at baseline was not associated with the rate of physical activity decline.

### Implications of all the available evidence

Our findings suggest that physical activity might be a modifiable risk factor for the prevention of dementia, primarily in the last years before dementia diagnosis. Therefore, our findings add more evidence to the body of work concerning the association between physical activity and dementia and the time course of this association and highlight the need for clinical trials that assess the timing of increased physical activity required to delay dementia in older adults.

## References

[1] Livingston G, Huntley J, Liu KY, Dementia prevention, intervention, and care: 2024 report of the *Lancet* standing Commission. Lancet 2024; 404: 572–628. 39096926 10.1016/S0140-6736(24)01296-0

[2] Petersen RC, Lopez O, Armstrong MJ, Practice guideline update summary: mild cognitive impairment [RETIRED]: report of the Guideline Development, Dissemination, and Implementation Subcommittee of the American Academy of Neurology. Neurology 2018; 90: 126–35. 29282327 10.1212/WNL.0000000000004826 PMC5772157

[3] Iso-Markku P, Kujala UM, Knittle K, Polet J, Vuoksimaa E, Waller K. Physical activity as a protective factor for dementia and Alzheimer’s disease: systematic review, meta-analysis and quality assessment of cohort and case-control studies. Br J Sports Med 2022; 56: 701–09. 35301183 10.1136/bjsports-2021-104981 PMC9163715

[4] Albers MW, Gilmore GC, Kaye J, At the interface of sensory and motor dysfunctions and Alzheimer’s disease. Alzheimers Dement 2015; 11: 70–98. 25022540 10.1016/j.jalz.2014.04.514 PMC4287457

[5] Nadkarni NK, Perera S, Snitz BE, Association of brain amyloid-β with slow gait in elderly individuals without dementia: influence of cognition and apolipoprotein E ε4 genotype. JAMA Neurol 2017; 74: 82–90. 27842173 10.1001/jamaneurol.2016.3474 PMC5735996

[6] Jack CR Jr, Andrews JS, Beach TG, Revised criteria for diagnosis and staging of Alzheimer’s disease: Alzheimer’s Association Workgroup. Alzheimers Dement 2024; 20: 5143–69. 38934362 10.1002/alz.13859 PMC11350039

[7] Perin S, Harrington KD, Lim YY, Amyloid burden and incident depressive symptoms in preclinical Alzheimer’s disease. J Affect Disord 2018; 229: 269–74. 29329059 10.1016/j.jad.2017.12.101

[8] Oveisgharan S, Wang T, Hausdorff JM, Bennett DA, Buchman AS. Motor and nonmotor measures and declining daily physical activity in older adults. JAMA Netw Open 2024; 7: e2432033. 39235807 10.1001/jamanetworkopen.2024.32033 PMC11378007

[9] Vermunt L, Sikkes SAM, van den Hout A, Duration of preclinical, prodromal, and dementia stages of Alzheimer’s disease in relation to age, sex, and APOE genotype. Alzheimers Dement 2019; 15: 888–98. 31164314 10.1016/j.jalz.2019.04.001 PMC6646097

[10] Oveisgharan S, Wang T, Barnes LL, Schneider JA, Bennett DA, Buchman AS. The time course of motor and cognitive decline in older adults and their associations with brain pathologies: a multicohort study. Lancet Healthy Longev 2024; 5: e336–45. 38582095 10.1016/S2666-7568(24)00033-3 PMC11129202

[11] Buchman AS, Boyle PA, Yu L, Shah RC, Wilson RS, Bennett DA. Total daily physical activity and the risk of AD and cognitive decline in older adults. Neurology 2012; 78: 1323–29. 22517108 10.1212/WNL.0b013e3182535d35 PMC3335448

[12] Verdelho A, Correia M, Ferro JM, Physical activity self-report is not reliable among subjects with mild vascular cognitive impairment: the AFIVASC study. J Alzheimers Dis 2022; 87: 405–14. 35275531 10.3233/JAD-215381

[13] Heil DP. Predicting activity energy expenditure using the Actical activity monitor. Res Q Exerc Sport 2006; 77: 64–80. 16646354 10.1080/02701367.2006.10599333

[14] Oveisgharan S, Yu L, Dawe RJ, Bennett DA, Buchman AS. Total daily physical activity and the risk of parkinsonism in community-dwelling older adults. J Gerontol A Biol Sci Med Sci 2020; 75: 702–11. 31046115 10.1093/gerona/glz111 PMC7328202

[15] McKhann GM, Knopman DS, Chertkow H, The diagnosis of dementia due to Alzheimer’s disease: recommendations from the National Institute on Aging-Alzheimer’s Association workgroups on diagnostic guidelines for Alzheimer’s disease. Alzheimers Dement 2011; 7: 263–69. 21514250 10.1016/j.jalz.2011.03.005 PMC3312024

[16] Ibrahim JG, Chu H, Chen LM. Basic concepts and methods for joint models of longitudinal and survival data. J Clin Oncol 2010; 28: 2796–801. 20439643 10.1200/JCO.2009.25.0654 PMC4503792

[17] Rizopoulos D The R package JMbayes for fitting joint models for longitudinal and time-to-event data Using MCMC. J Stat Softw 2016; 72: 1–46.

[18] Tan X, Shiyko MP, Li R, Li Y, Dierker L. A time-varying effect model for intensive longitudinal data. Psychol Methods 2012; 17: 61–77. 22103434 10.1037/a0025814 PMC3288551

[19] Wang Z, Emmerich A, Pillon NJ, Genome-wide association analyses of physical activity and sedentary behavior provide insights into underlying mechanisms and roles in disease prevention. Nat Genet 2022; 54: 1332–44. 36071172 10.1038/s41588-022-01165-1 PMC9470530

[20] Bellenguez C, Küçükali F, Jansen IE, New insights into the genetic etiology of Alzheimer’s disease and related dementias. Nat Genet 2022; 54: 412–36. 35379992 10.1038/s41588-022-01024-z PMC9005347

[21] WHO. Global action plan on the public health response to dementia 2017–2025. 2017. https://www.who.int/publications/i/item/global-action-plan-on-the-public-health-response-to-dementia-2017—2025 (accessed April 20, 2025).

[22] Stern Y, Albert M, Barnes CA, Cabeza R, Pascual-Leone A, Rapp PR. A framework for concepts of reserve and resilience in aging. Neurobiol Aging 2023; 124: 100–03. 36653245 10.1016/j.neurobiolaging.2022.10.015 PMC10424718

[23] Wrann CD, White JP, Salogiannnis J, Exercise induces hippocampal BDNF through a PGC-1α/FNDC5 pathway. Cell Metab 2013; 18: 649–59. 24120943 10.1016/j.cmet.2013.09.008 PMC3980968

[24] Buchman AS, Yu L, Wilson RS, Physical activity, common brain pathologies, and cognition in community-dwelling older adults. Neurology 2019; 92: e811–22. 30651386 10.1212/WNL.0000000000006954 PMC6396972

[25] Lin J-C, Chen I-H, Cheng F-Y. Review articles (meta-analyses) effects of walking on cognitive function in individuals with mild cognitive impairment: a systematic review and meta-analysis. BMC Geriatr 2023; 23: 500. 37605156 10.1186/s12877-023-04235-z PMC10441758

[26] Ciria LF, Román-Caballero R, Vadillo MA, An umbrella review of randomized control trials on the effects of physical exercise on cognition. Nat Hum Behav 2023; 7: 928–41. 36973359 10.1038/s41562-023-01554-4

[27] Cao Y, Rajan SS, Wei P. Mendelian randomization analysis of a time-varying exposure for binary disease outcomes using functional data analysis methods. Genet Epidemiol 2016; 40: 744–55. 27813215 10.1002/gepi.22013 PMC5123677

