# Supplementary material for: Repeated measures of physical activity before dementia diagnosis in community-dwelling older adults: a longitudinal study
Source: Lancet Healthy Longev. Author manuscript; Available in PMC 2026 Apr 8. (PMC13059499; doi:10.1016/j.lanhl.2026.100824)
Supplement: 1 [file NIHMS2157676-supplement-1.pdf]

THE LANCET
Healthy Longevity

Supplementary appendix

This appendix formed part of the original submission and has been peer reviewed.
We post it as supplied by the authors.

Supplement to: Oveisgharan S, Yang J, Wang T, et al. Repeated measures of physical activity before dementia diagnosis in community-dwelling older adults: a longitudinal study. *Lancet Healthy Longev* 2026. https://doi.org/10.1016/j.lanhl.2026.100824

Content | **Pages**

Content	Pages
**Table e-1. Comparison of the included and excluded participants.**	**3**
**eMethods**	**4-6**
**eResults**	**7**
**Figure e-1. Comparison of joint modeling and time-varying Cox model.**	**8**
**Table e-2. Association of repeated measures of physical activity with the risk of dementia in different subgroups of the participants.**	**9**
**Table e-3. Association of physical activity with the risk of dementia when examining rate of change in the level of physical activity or longitudinal levels of activity counts per hour instead of longitudinal levels of physical activity.**	**10**
**Table e-4. Association of physical activity at baseline vs. at years 2-6 of follow-up with the risk of AD 7 or more years after baseline.**	**11**
**Figure e-2. The trajectory of physical activity derived from a time-varying effects model.**	**12**
**Figure e-3. The retrospective association of dementia with the repeated measures of physical activity prior to dementia after controlling for covariates.**	**13**
**Table e-5. The linear mixed effects models examining the association between mild cognitive impairment (MCI) or global cognition score at baseline with longitudinal changes of physical activity.**	**14**

Figure e-4. A spaghetti plot of longitudinal levels of physical activity of 25 randomly selected participants with either MCI or no cognitive impairment at baseline. **15**

Figure e-5. Testing a non-linear association between MCI status at baseline and the repeated measures of physical activity. **16**

Table e-6. The relation with AD of 16 genetic variants associated with physical activity. **17**

Table e-7. Mendelian randomization estimates of the association between genetic susceptibility to high or low physical activity and the risk of AD. **18**

Table e-8. The relation with physical activity of 58 genetic variants associated with Alzheimer's disease. **19-23**

Table e-9. Mendelian randomization estimates of the association between genetic susceptibility to AD with the level of physical activity. **24**

Table e-1. Comparison of the included and excluded participants.

Measure	Included (n=972)	Excluded-no physical activity assessment (n=197)	Excluded-No valid follow-up (N=229)
**Demographic**			
Age at baseline (years), mean (SD)	80.5 (7.3)***	84.9 (7.6)	82.1 (7.4)
Women, n (%)	745 (76.7)	142 (72.1)	182 (79.5)
Race/ethnicity			
Latino, n (%)	27 (2.8)	5 (2.5)	6 (2.6)
Non-Latino White, n (%)	892 (91.8)	176 (89.3)	204 (89.1)
Non-Latino Black, n (%)	48 (4.9)	12 (6.1)	18 (7.9)
Other[a], n (%)	5 (0.5)	4 (2.0)	1 (0.4)
Years of education, mean (SD)	15.1 (3.0)*	14.9 (3.2)	14.7 (3.0)
**Clinical characteristics at baseline**			
Number of vascular risk factors (hypertension, diabetes mellitus, smoking), median (IQR)	1.0 (1.0–2.0)	1.0 (1.0–2.0)	1.0 (1.0–2.0)
Body mass index, mean (SD)	27.4 (5.4)*	26.1 (4.7)	27.3 (5.4)
History of stroke, n (%)	91 (10.4)*	30 (17.2)	29 (13.4)
ApolipoproteinE ε4 allele , n (%)	200 (21.1)**	61 (33.5)	45 (23.1)
Number of depressive symptoms, median (IQR)	0.0 (0.0–1.0)***	1.0 (0.0–2.0)	1.0 (0.0–2.0)
Purpose in life, median (IQR)	3.8 (3.5–4.0)***	3.5 (3.2–3.8)	3.6 (3.2–3.9)
Social activity, median (IQR)	2.67 (2.33–3.17)***	2.25 (1.83–2.67)	2.50 (2.00–2.83)
Cognitive activity, median (IQR)	3.29 (2.86–3.57)***	2.86 (2.29–3.29)	3.14 (2.57–3.57)
Dementia, n (%)	0 (0)***	106 (60.6)	4 (1.8)

For comparing characteristics across the three groups, analysis of variance (continuous variables), Chi-square (categorical variables), and Kruskal-Wallis (ordinal variables) tests were used.

[a]Races and ethnicities with a frequency less than 5 are included under "other".

*, $p<0.05$; **, $p<0.01$; ***, $p<0.001$.

eMETHODS

Other covariates

In the current study a dichotomous variable was used to indicate the presence of one or more *APOE ε4* allele, At MAP enrollment, the participants provided their date of birth, sex, race, ethnicity, and years of education by self-report. Self-report questions were also used to obtain vascular risk factors status (hypertension, diabetes mellitus, and smoking), which were summarized by a composite variable indicating the number of present risk factors. Body mass index was calculated using weight and height measured annually by a research assistant. The presence of stroke was adjudicated by a clinician based on the history of related symptoms and the neurological examination findings. Structured questionnaires were adapted for measuring the following covariates: depressive symptoms (Center for Epidemiologic Studies Depression scale), purpose in life (Ryff's scales of Psychological Well-Being), participation in social activities (a summary of 6 questions asking about participation in six social activities[1]), and participation in cognitive activities (a summary of seven questions asking about participation in seven cognitive activities[2]). Sequencing of the *apolipoprotein E (APOE)* gene was performed using DNA extracted from blood or brain tissue. In the current study a dichotomous variable was used to indicate the presence of one or more *APOE ε4* allele[3].

A 26-item modified motor portion of the Unified Parkinson's Disease Rating Scale was used for the collection of the four parkinsonian signs data: bradykinesia, rigidity, tremor, and parkinsonian gait. Trained nurse clinicians examined the participants annually to collect the parkinsonian data, which were summarized by a dichotomous variable indicating the presence of two or more parkinsonian signs. Moreover, the 26 items' scores were summed and scaled to create a global composite score indicating the severity of parkinsonism[4]. Our prior study showed that the nurses-collected data were reliable and had a high degree of agreement with the examination done by a movement disorder specialist[5].

Mendelian Randomization Analysis

Data sources and study design
To further investigate the relationship between physical activity and dementia, we conducted a bi-directional two-sample MR analysis to assess the potential causal effect of physical activity on AD dementia risk and potential reverse causation. We utilized publicly available summary statistics from genome-wide association studies (GWAS). Data related to physical activity were obtained from the UK Biobank (GCP ID: GCP000358)[6], and data related to AD were accessed via the EBI GWAS Catalog (accession no. GCST90027158)[7]. These datasets were derived from independent, non-overlapping studies of adults with European ancestry.

Quality Control and Instrument Selection
Prior to analysis, we performed rigorous quality control on the summary data. We restricted the dataset to biallelic variants with

standard rsID identifiers and complete summary statistics (beta, standard error, p-value, and allele frequency). We removed duplicated variants, SNPs with out-of-bounds allele frequencies (≤0 or ≥1), and excluded all ambiguous palindromic variants (A/T or C/G) to prevent strand mismatch errors.

We identified instrumental variables (IVs) for physical activity using a genome-wide significance threshold of $p < 5 \times 10^{-8}$. To ensure independence among instruments, selected SNPs were clumped using a linkage disequilibrium (LD) threshold of $r^2 < 0.001$ within a 10,000 kb window, utilizing the 1000 Genomes Project European reference panel. We assessed the strength of the genetic instruments by calculating the F-statistic for each SNP ($F = \beta^2/SE^2$). An F-statistic greater than 10 was considered indicative of sufficient instrument strength to minimize weak instrument bias. Equivalent selection and QC procedures were applied to identify genetic instruments for AD dementia for the reverse direction analysis.

Data harmonization and MR analysis
Prior to analysis data harmonization was conducted using the *harmonise_data* function in the *TwoSampleMR* R package (R version 4.4.1) to ensure consistent alignment of effect alleles between the exposure and outcome datasets. The primary analysis was conducted using a multiplicative random effects inverse-variance weighted (IVW) model. To assess the robustness of the findings and detect potential pleiotropy, we employed sensitivity analyses including the MR-Egger regression and weighted median methods.

Statistical analyses-Joint modeling

We analyzed longitudinal physical activity measures and incident dementia with a shared-parameter joint model. In this framework, each participant's activity trajectory is described by a linear mixed-effects sub-model with random intercepts and slopes, while a proportional-hazards sub-model relates the continuously updated, person-specific level of physical activity to dementia risk; both components are fitted simultaneously via Bayesian Markov chain Monte Carlo, rather than sequentially estimating a mixed model and then feeding prediction into a separate Cox model. This single-step estimation minimizes regression-to-the-mean bias and increases power compared with a conventional time-varying Cox model[8], which treats each observed activity value as constant until the next visit and therefore misrepresents the typical decline of physical activity seen in older adults[9]. Figure e-1 illustrates the difference: for a participant assessed at baseline, year 2, and year 5 over eight years of follow-up, the joint model reconstructs a smooth trajectory that continuously informs the hazard, whereas the time-varying Cox model uses step functions that overestimate activity between visits. The joint modeling was implemented using the JMbayes R package (R version 4.4.1)[10]. For comparison, we examined time-invariant Cox models to examine the associations of a single measurement of physical activity at different time points in relation to incident dementia. All joint and comparison models were adjusted for age, sex, and education.

References:

1 Buchman AS, Boyle PA, Wilson RS, Fleischman DA, Leurgans S, Bennett DA. Association between late-life social activity and motor decline in older adults. *Arch Intern Med* 2009; **169**: 1139–46.

2 Wilson RS, Segawa E, Boyle PA, Bennett DA. Influence of late-life cognitive activity on cognitive health. *Neurology* 2012; **78**: 1123–9.

3 Oveisgharan S, Buchman AS, Yu L, *et al.* APOE ε2ε4 genotype, incident AD and MCI, cognitive decline, and AD pathology in older adults. *Neurology* 2018; **90**: e2127–34.

4 Oveisgharan S, Yu L, Dawe RJ, Bennett DA, Buchman AS. Total daily physical activity and the risk of parkinsonism in community-dwelling older adults. *The journals of gerontology Series A, Biological sciences and medical sciences* 2019; **75**: 702–11.

5 Bennett DA, Shannon KM, Beckett LA, Goetz CG, Wilson RS. Metric properties of nurses' ratings of parkinsonian signs with a modified Unified Parkinson's Disease Rating Scale. *Neurology* 1997; **49**: 1580–7.

6 Wang Z, Emmerich A, Pillon NJ, *et al.* Genome-wide association analyses of physical activity and sedentary behavior provide insights into underlying mechanisms and roles in disease prevention. *Nat Genet* 2022; **54**: 1332–44.

7 Bellenguez C, Küçükali F, Jansen IE, *et al.* New insights into the genetic etiology of Alzheimer's disease and related dementias. *Nat Genet* 2022; **54**: 412–36.

8 Ibrahim JG, Chu H, Chen LM. Basic concepts and methods for joint models of longitudinal and survival data. *J Clin Oncol* 2010; **28**: 2796–801.

9 Oveisgharan S, Wang T, Hausdorff JM, Bennett DA, Buchman AS. Motor and Nonmotor Measures and Declining Daily Physical Activity in Older Adults. *JAMA Netw Open* 2024; **7**: e2432033.

10 Rizopoulos D. The R Package JMbayes for Fitting Joint Models for Longitudinal and Time-to-Event Data Using MCMC. *J Stat Soft* 2016; **72**: 1–46.

eResults

Sensitivity analyses of the joint model's findings.

The joint model's findings about the association of longitudinal levels of physical activity with the risk of dementia did not change when the joint models were examined separately in females and males, in participants with and without APOE ε4, or when participants with parkinsonism were excluded (**Table e-2**). Furthermore, replacing the mixed effects model-derived person-specific levels of physical activity with the model-derived person-specific rates of change in physical activity or replacing total daily activity counts with activity counts per hour did not change the association between physical activity and incident dementia (**Table e-3**).

Figure e-1. Comparison of joint modeling and time-varying Cox model.

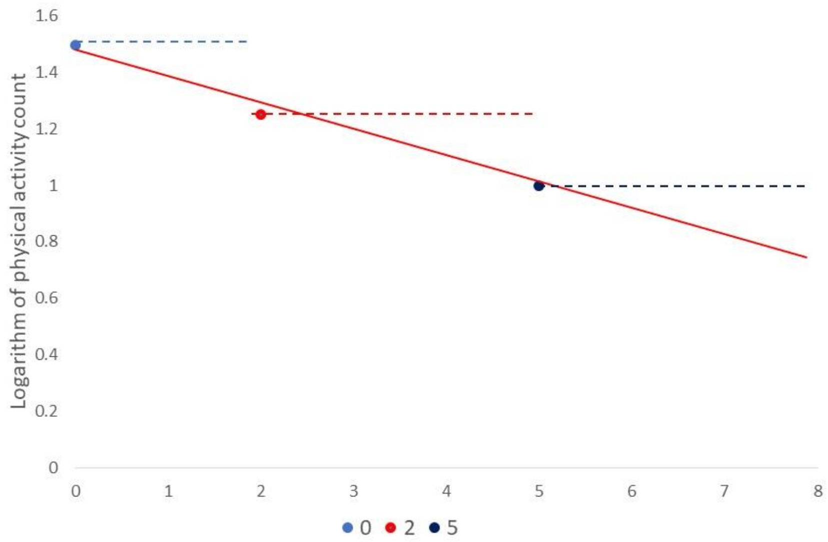

The figure illustrates a joint model vs a time-varying Cox model in examining the association of physical activity with the risk of dementia using data of a participant with 3 measurements of physical activity (the blue dots) during 8 years of follow up, at baseline (red dot), follow up years 2 (blue dot) and 5 (black dot). The joint model estimates a person-specific line of the level of physical activity over 8 years of follow up for this participant, based on the 3 measurements of physical activity, and uses these estimated levels in the association with the risk of dementia. The time-varying Cox model uses this participant's baseline level of physical activity as the level of physical activity from baseline to before year 2, the year 2 level of physical activity as the level of physical activity from year 2 to before year 5, and the year 5 level of physical activity as the level for the rest of follow up.

Table e-2. Association of repeated measures of physical activity with the risk of dementia in different subgroups of the participants.

Model	Subgroup of the participants	Association of the repeated measures of physical activity with the risk of dementia: HR (95% confidence interval), p-value
1	Females	0.81 (0.71 – 0.93), 0.0033
2	Males	0.66 (0.46 – 0.92), 0.0091
3	With *ApolipoproteinE ε4 allele*	0.74 (0.56 – 0.96), 0.021
4	Without *ApolipoproteinE ε4 allele*	0.76 (0.66 – 0.89), <0.0001
5	After exclusion of participants with parkinsonism	0.80 (0.68 – 0.94), 0.0065

Table e-3. Association of physical activity with the risk of dementia when examining rate of change in the level of physical activity or longitudinal levels of activity counts per hour instead of longitudinal levels of physical activity.

Model	Physical activity measure	Association with the risk of dementia: HR (95% confidence interval), p-value
Reference-A	Mixed-effects submodel-derived person-specific longitudinal levels of the average activity counts per day	0.78 (0.69–0.88), <0.0001
B	Mixed-effects submodel-derived person-specific longitudinal rates of change in the average activity counts per day	0.78 (0.67–0.90), <0.0001
C	Mixed-effects submodel-derived person-specific longitudinal levels of the activity counts per hour	0.65 (0.52–0.80), <0.0001

In the joint models, we can use either the mixed-effects submodel-derived longitudinal levels or longitudinal slopes of change of the outcome (physical activity measure in the current study) as the time-varying covariate in the proportional-hazards submodel. In three separate joint models, we examined the association of longitudinal measurements of physical activity with incident dementia. In the models A (A was the main model in the manuscript) and B, the outcome of the mixed-effects submodel was the average activity counts per day. However, in the model A the mixed-effects submodel-derived person-specific longitudinal levels of the average activity counts per day was used as the time-varying covariate in the proportional-hazards sub-model while in the model B the time-varying covariate was the longitudinal slopes of change of the average activity counts per day. In the model C, the outcome of the mixed-effects submodel was the activity counts per hour, and the proportional-hazards submodel time-varying covariate was the mixed-effects submodel-derived person-specific longitudinal levels of the activity counts per hour. The analyses show that physical activity was associated with a lower risk of dementia irrespective of the examined model.

Table e-4. Association of physical activity at baseline vs. at years 2-6 of follow-up with the risk of dementia 7 or more years after baseline.

Time of physical activity measurement	Number of incident dementia	Number of censored	Association with dementia: HR (95% confidence interval), p-value
Baseline	122	686	1.00 (0.69 – 1.45), 0.99
Year 2	98	669	0.61 (0.43 – 0.86), 0.0050
Year 4	94	591	0.61 (0.44 – 0.85), 0.0032
Year 6	75	493	0.55 (0.37 – 0.80), 0.0021

In 4 separate Cox models, we examined the association of physical activity, measured either at baseline or in years 2, 4, 6, with the risk of dementia that had occurred after 7 years of follow-up. Because physical activity was not measured for all the participants at every year of follow-up, fewer participants were included in the models that examined physical activity at years 2-6.

Figure e-2. The trajectory of physical activity derived from a time-varying effects model.

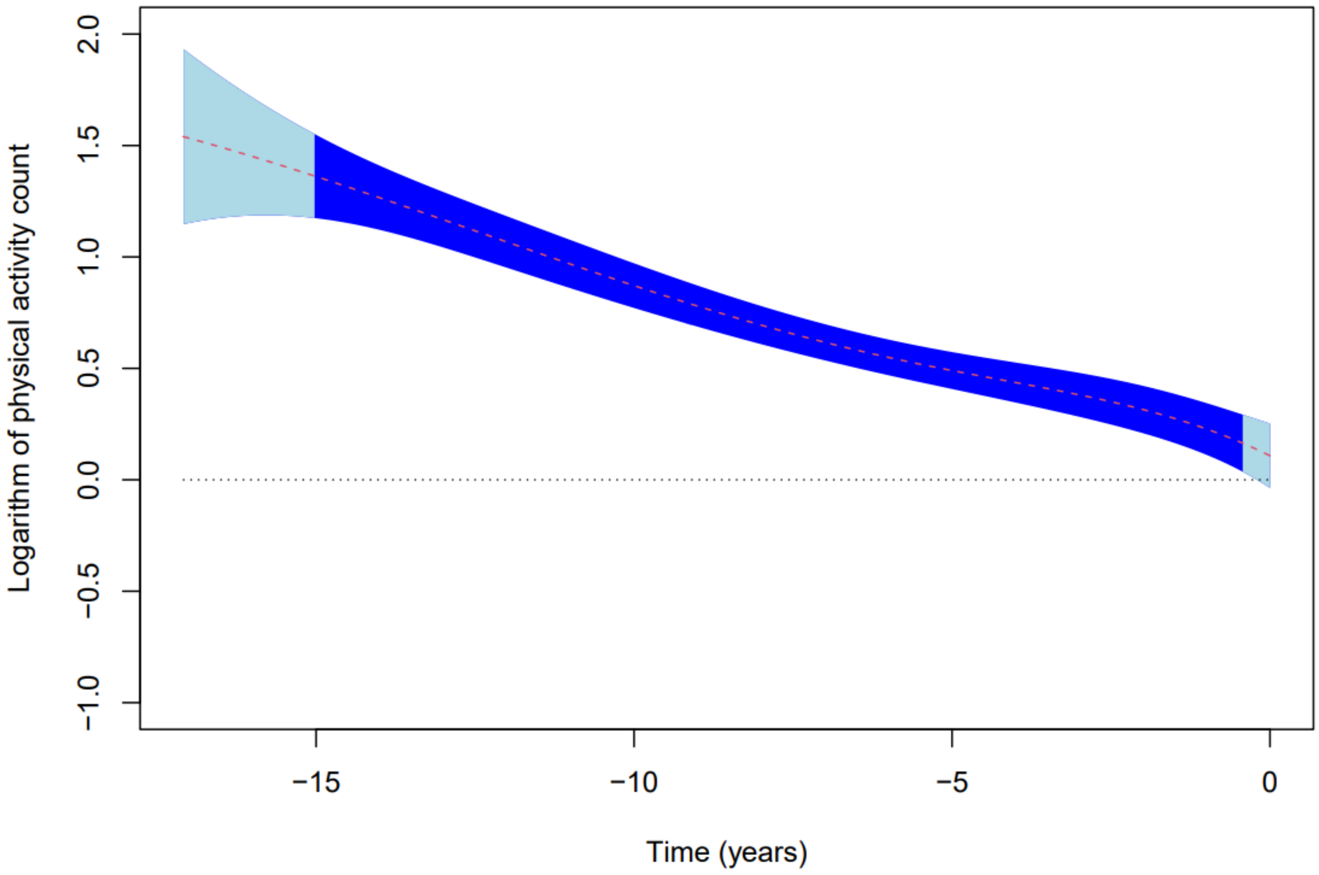

Dark blue indicates the area of the trajectory with the densest physical activity data, and light blue indicates tails where physical activity data are sparser.

The figure indicates that on average physical activity is declining in older adults.

Figure e-3. The retrospective association of dementia with the repeated measures of physical activity prior to dementia after controlling for covariates.

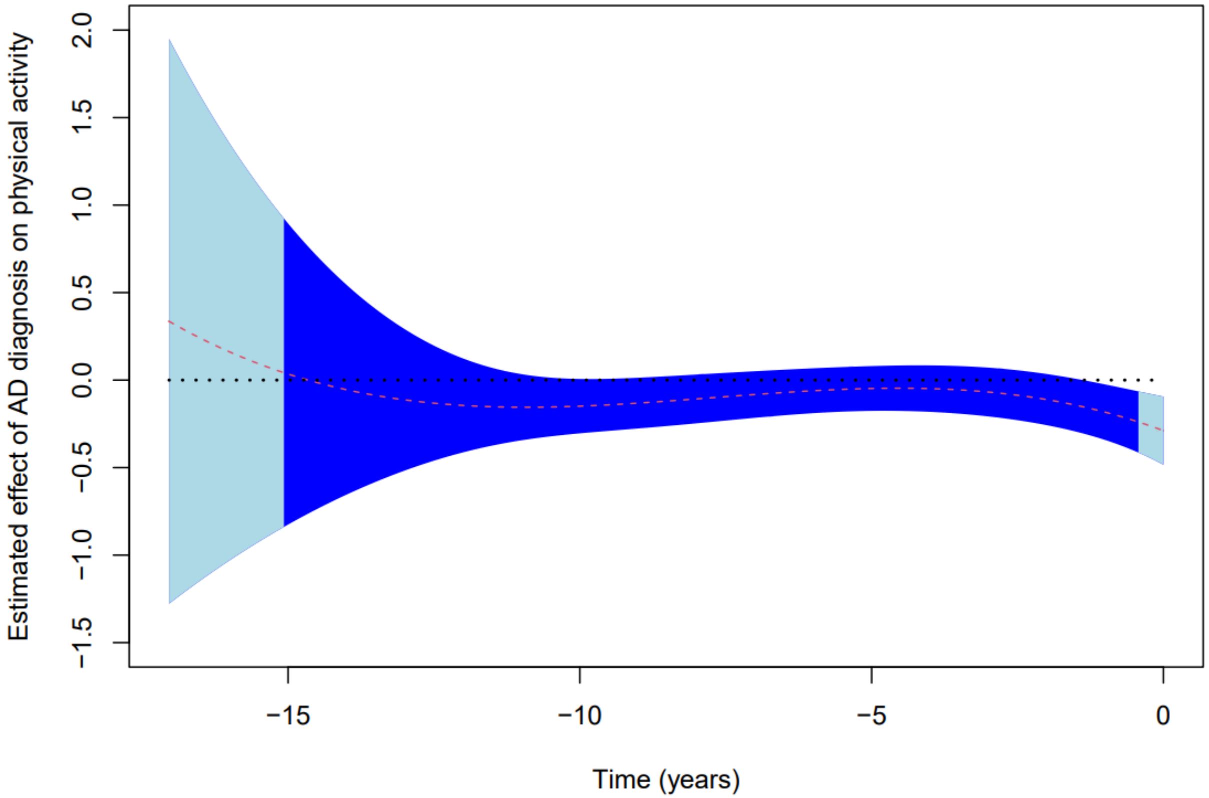

The figure indicates that physical activity is not different between participants with and without dementia until 1.8 years before dementia diagnosis when incipient dementia cases have lower levels of physical activity. The model was adjusted for age, sex, education, vascular risk factors, body mass index, stroke, depressive symptoms, purpose in life, cognitive and social activity, MCI, *APOE ε4*. The red dotted line is the point estimate of the association between dementia and physical activity while the black dotted line is the line of zero indicating no difference between participants with and without dementia in physical activity. When the red dotted line and its confidence interval (the shading areas) cover the black dotted line of zero, it indicates that there is no association between dementia and physical activity.

Table e-5. The linear mixed effects models examining the association between mild cognitive impairment (MCI) or global cognition score at baseline with longitudinal changes of physical activity.

Model	Model term	Slope of physical activity	Baseline level of physical activity
		Estimate (SE), p-value	
1	MCI	0.016 (0.011), 0.18	-0.138 (0.047), 0.0036
2	Global cognition score	0.006 (0.010), 0.52	0.104 (0.043), 0.017

In two separate linear mixed effects models, we examined the association of MCI or global cognition score at baseline with longitudinal changes of physical activity. The fixed model terms were either MCI or global cognition score in addition, to age at baseline, sex, education, time, an interaction of time with the other model terms. The random effect model terms were intercept and time.

Figure e-4. A spaghetti plot of longitudinal levels of physical activity of 25 randomly selected participants with either MCI or no cognitive impairment at baseline.

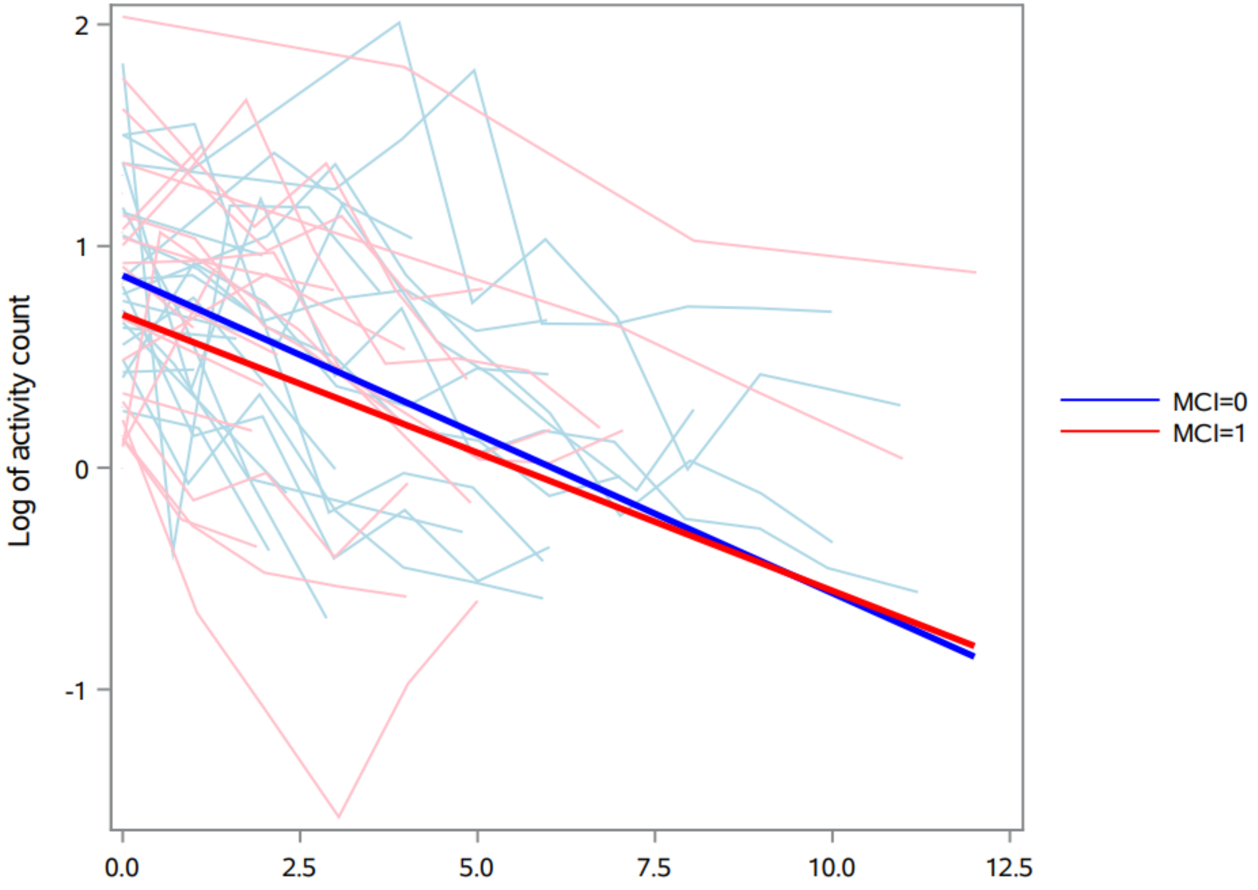

The red lines show years of physical activity levels of participants with MCI at baseline, and the blue lines illustrate the physical activity levels of participants with no cognitive impairment at baseline. Time 0 indicates baseline. The superimposed thicker lines indicate the average linear changes of physical activity in the two groups of participants.

Figure e-5. Testing a non-linear association between MCI status at baseline and the repeated measures of physical activity.

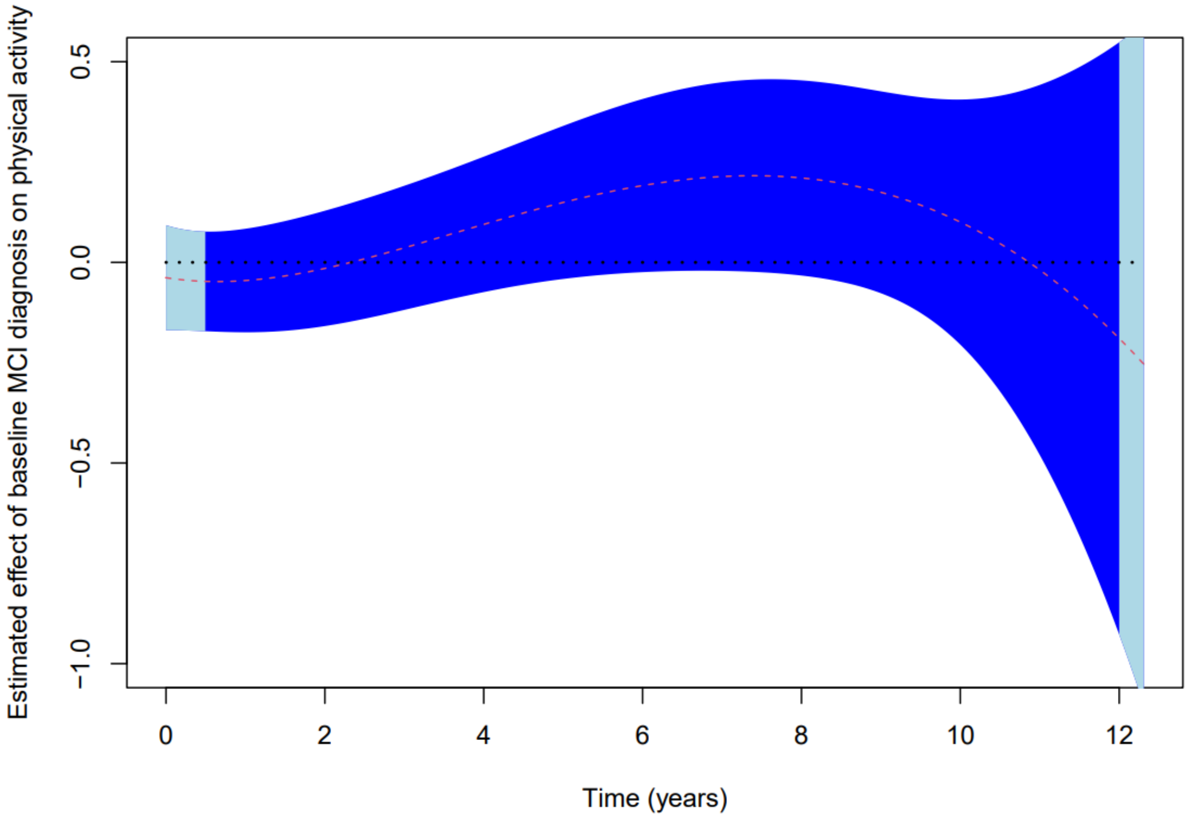

187 participants with MCI at baseline were matched with 187 participants with no cognitive impairment at baseline by age at baseline (±3 years), sex, and duration of follow-up (±3 years). Then, the association between MCI status at baseline with the repeated measures of physical activity was tested using a time-varying effects model. The figure indicates that baseline MCI status is not associated with the repeated levels of physical activity as the confidence interval (the shaded area) always included 0 (the black dotted line). The red dotted line is the point estimate of the association between MCI and physical activity while the black dotted line is the line of zero indicating no difference between participants with and without MCI in physical activity. When the red dotted line and its confidence interval (the shading areas) cover the black dotted line of zero, it indicates that there is no association between MCI and physical activity.

Table e-6. The relation with AD of 16 genetic variants associated with physical activity.

Variant ID	EA	OA	Association with physical activity						Association with AD				
			EAF	Sample size	Estimate	SE	P-value	F	EAF	Sample size		OR (95% CI)	P-value
										AD	Control		
rs1160545	T	C	0.4025	483768	0.0249	0.0041	1.73E-09	36.9	0.3888	85934	401577	1.01 (0.99-1.03)	0.18
rs12357890	A	G	0.4431	483768	0.0225	0.0041	4.77E-08	30.1	0.4553	84465	394472	1 (0.99-1.02)	0.81
rs13201721	T	C	0.7364	522597	0.0255	0.004	1.83E-10	40.6	0.7348	85934	401577	0.99 (0.98-1.01)	0.51
rs1625595	T	C	0.4748	592552	-0.0213	0.0032	1.90E-11	44.3	0.4761	85934	401577	0.99 (0.97-1.01)	0.20
rs1691471	T	C	0.3759	483768	0.0379	0.0042	1.73E-19	81.4	0.3758	85934	401577	1 (0.98-1.02)	0.94
rs334954	A	G	0.3033	483768	0.0241	0.0044	4.85E-08	30	0.3181	85934	401577	1 (0.98-1.01)	0.75
rs370935521	T	C	0.0926	448902	0.0434	0.0075	8.25E-09	33.5	0.0973	84465	394472	1.02 (0.99-1.05)	0.18
rs385301	T	C	0.2637	461744	-0.0284	0.0047	1.60E-09	36.5	0.2572	85934	401577	1.03 (1.01-1.05)	0.0024
rs4352559	T	C	0.4964	599961	0.0180	0.0032	1.65E-08	31.6	0.4945	85934	401577	0.99 (0.97-1)	0.08
rs4865512	A	G	0.6124	483768	0.0240	0.0042	7.68E-09	32.7	0.6163	85934	401577	1 (0.99-1.02)	0.72
rs568546	T	C	0.5206	483768	0.0237	0.0041	5.89E-09	33.4	0.522	85934	401577	0.99 (0.98-1.01)	0.31
rs6427178	A	G	0.5318	484633	0.0229	0.0041	1.71E-08	31.2	0.5219	85934	401577	1.01 (0.99-1.03)	0.26
rs7613360	T	C	0.3963	483768	-0.0247	0.0042	2.77E-09	34.6	0.3903	85934	401577	1 (0.99-1.02)	0.66
rs9420	A	G	0.3367	604253	-0.0184	0.0033	3.33E-08	31.1	0.3207	85934	401577	1 (0.99-1.02)	0.58
rs9881048	A	C	0.1914	483768	-0.0288	0.0052	2.54E-08	30.7	0.1981	85934	401577	1 (0.98-1.02)	0.92
rs9903845	A	C	0.3104	604232	-0.0200	0.0034	6.05E-09	34.6	0.3107	85934	401577	1 (0.98-1.01)	0.75

EA: Effective allele; OA: Other allele; EAF: Effective allele frequency;

Table e-7. Mendelian randomization estimates of the association between genetic susceptibility to high or low physical activity and the risk of AD.

Method	Association with AD	
	OR (95% CI)	P-value
**Inverse variance weighted**	0.97 (0.79-1.18)	0.76
**Weighted median**	1.01 (0.79-1.27)	0.96
**MR Egger**	1.24 (0.48-3.17)	0.66
**Simple mode**	0.98 (0.65-1.46)	0.90
**Weighted mode**	0.98 (0.68-1.41)	0.92

Table e-8. The relation with physical activity of 58 genetic variants associated with Alzheimer's disease.

			Association with AD						Association with physical activity				
**Variant ID**	**Effect allele**	**Other allele**	**EAF**	**No of AD**	**No of Ctrl**	**OR (95%CI)**	**P-Value**	**F**	**EAF**	**Sample size**	**Estimate**	**SE**	**P-value**
**rs10792832**	A	G	0.36	85934	401577	0.9 (0.89-0.91)	6.33E-36	158.0	0.36	604253	0.001	0.0033	0.76
**rs10838702**	T	G	0.40	85934	401577	1.06 (1.04-1.08)	7.84E-12	46.3	0.38	602216	-0.0023	0.0033	0.48
**rs10948358**	T	G	0.37	85934	401577	1.05 (1.03-1.07)	9.94E-09	32.6	0.38	483768	0.001	0.0042	0.82
**rs11218343**	T	C	0.96	85934	401577	1.18 (1.13-1.23)	1.01E-14	59.7	0.97	581324	-0.0024	0.0093	0.80
**rs112481437**	A	G	0.028	85665	400902	1.34 (1.27-1.42)	4.86E-26	111.5	0.029	480970	-8.00E-04	0.0128	0.95
**rs113706587**	A	G	0.110	85934	401577	1.1 (1.07-1.13)	3.38E-12	48.4	0.10	482750	-0.015	0.0068	0.028
**rs1140239**	T	C	0.379	84465	394472	0.94 (0.93-0.96)	4.61E-12	47.4	0.40	483768	0.0073	0.0041	0.076
**rs1160983**	A	G	0.030	85665	400902	0.58 (0.55-0.62)	4.99E-83	371.8	0.031	470146	-0.0148	0.012	0.22
**rs117310449**	T	C	0.017	85502	400497	2.16 (2.02-2.31)	3.26E-115	519.1	0.012	482668	-0.0125	0.0187	0.51
**rs117618017**	T	C	0.14	85934	401577	1.12 (1.09-1.15)	1.75E-21	90.2	0.14	482750	-0.0046	0.0059	0.44

**rs11769559**	T	C	0.59	84196	393797	1.07 (1.05-1.09)	4.00E-16	66.9	0.59	448902	-9.00E-04	0.0043	0.83
**rs12151021**	A	G	0.34	85665	400902	1.11 (1.09-1.13)	4.09E-30	128.7	0.33	482750	-0.0026	0.0044	0.56
**rs12444183**	A	G	0.39	85934	401577	0.94 (0.93-0.96)	2.21E-12	49.5	0.37	602447	-0.0012	0.0033	0.71
**rs12590654**	A	G	0.33	85934	401577	0.93 (0.92-0.95)	2.08E-15	63.4	0.34	469128	0.0077	0.0044	0.081
**rs137945009**	A	C	0.44	85665	400902	0.95 (0.93-0.96)	1.72E-10	41.1	0.43	483768	0.0016	0.0041	0.69
**rs143332484**	T	C	0.012	85665	400902	1.4 (1.3-1.5)	6.04E-19	79.2	0.010	477754	0.0075	0.0205	0.72
**rs1582763**	A	G	0.37	85934	401577	0.92 (0.9-0.93)	1.65E-24	104.8	0.36	604123	0.0033	0.0033	0.32
**rs1693551**	T	C	0.53	85934	401577	0.96 (0.94-0.97)	1.79E-08	32.1	0.54	604247	-0.0032	0.0032	0.30
**rs17125924**	A	G	0.91	85934	401577	0.92 (0.89-0.94)	5.82E-10	38.5	0.90	606025	0.003	0.0054	0.58
**rs190651665**	T	G	0.97	85665	400902	1.17 (1.11-1.23)	8.37E-10	37.8	0.97	481650	-0.0023	0.0114	0.84
**rs2070902**	T	C	0.25	85934	401577	0.95 (0.93-0.97)	1.05E-08	32.6	0.25	605112	0.0027	0.0037	0.46
**rs2154481**	T	C	0.52	85934	401577	1.05 (1.03-1.07)	1.02E-09	37.2	0.52	604254	0.004	0.0032	0.21

**rs2526377**	A	G	0.56	85934	401577	1.05 (1.03-1.06)	4.11E-08	30.4	0.56	483768	0.0018	0.0041	0.67
**rs2830489**	T	C	0.28	85934	401577	0.95 (0.93-0.96)	1.72E-09	36.1	0.28	603234	-0.0032	0.0036	0.37
**rs28577986**	A	G	0.26	83994	388096	1.07 (1.05-1.1)	3.38E-10	39.5	0.28	477917	0.0074	0.0045	0.10
**rs34173062**	A	G	0.081	85665	400902	1.12 (1.09-1.16)	2.93E-12	48.8	0.072	477917	0.006	0.0084	0.47
**rs35472547**	T	G	0.16	84465	394472	0.91 (0.89-0.93)	4.27E-17	71.3	0.19	479952	-0.0095	0.0052	0.069
**rs365653**	A	G	0.89	85665	400902	1.29 (1.26-1.33)	1.85E-68	307.4	0.88	482750	0.0066	0.0064	0.31
**rs4292**	T	C	0.62	85934	401577	1.07 (1.05-1.09)	3.47E-16	67.1	0.63	483768	0.0048	0.0042	0.25
**rs4485362**	T	G	0.54	85934	401577	1.05 (1.03-1.06)	2.00E-08	31.1	0.53	482750	2.00E-04	0.0041	0.96
**rs4690197**	T	G	0.20	85934	401577	0.94 (0.92-0.96)	8.39E-09	33.0	0.21	483768	0.0028	0.005	0.57
**rs4714447**	T	C	0.34	85934	401577	1.06 (1.04-1.08)	1.73E-11	45.2	0.35	483768	0.0086	0.0043	0.045
**rs4734295**	A	G	0.54	85934	401577	0.95 (0.94-0.97)	1.98E-09	36.1	0.54	606016	0.0027	0.0032	0.39
**rs563366239**	A	G	0.99	85665	400902	1.35 (1.24-1.46)	3.28E-12	48.4	0.99	448902	-0.0118	0.0209	0.57

**rs57402520**	A	G	0.12	84465	394472	1.09 (1.07-1.12)	6.59E-13	51.6	0.12	483768	-0.0135	0.0063	0.033
**rs5848**	T	C	0.29	85934	401577	1.07 (1.05-1.09)	1.76E-12	49.3	0.29	593294	-0.0035	0.0036	0.34
**rs593742**	A	G	0.71	85934	401577	1.06 (1.04-1.08)	1.04E-11	45.9	0.69	604255	-0.0094	0.0034	0.0058
**rs6014724**	A	G	0.91	85934	401577	1.12 (1.09-1.16)	4.84E-16	65.8	0.91	602544	-6.00E-04	0.0057	0.91
**rs61762319**	A	G	0.97	85934	401577	0.87 (0.82-0.91)	2.14E-08	31.4	0.97	476737	-0.0066	0.0122	0.59
**rs62375397**	T	C	0.21	85934	401577	1.08 (1.06-1.1)	8.58E-14	55.7	0.22	483768	-0.0012	0.0049	0.81
**rs6586028**	T	C	0.80	85934	401577	1.08 (1.06-1.1)	1.33E-14	59.0	0.79	483768	0.0047	0.005	0.35
**rs6656401**	A	G	0.19	85934	401577	1.13 (1.11-1.16)	2.84E-33	145.2	0.20	601789	-0.0026	0.004	0.52
**rs67250450**	T	C	0.79	85934	401577	1.06 (1.04-1.08)	2.02E-08	31.2	0.80	481733	0.0019	0.0051	0.71
**rs6733839**	T	C	0.39	85934	401577	1.18 (1.16-1.2)	6.48E-90	402.9	0.39	482750	0.0023	0.0042	0.59
**rs6846529**	T	C	0.72	85934	401577	0.93 (0.92-0.95)	1.25E-13	54.7	0.74	491541	5.00E-04	0.0041	0.89
**rs7068231**	T	G	0.40	85934	401577	0.95 (0.94-0.97)	6.79E-09	33.6	0.40	604246	3.00E-04	0.0033	0.92
**rs7225002**	A	G	0.60	85934	401577	1.06 (1.04-1.07)	1.78E-10	40.9	0.59	452635	-0.0143	0.0042	0.0007

**rs73223431**	T	C	0.37	85934	401577	1.07 (1.05-1.09)	5.34E-15	61.0	0.37	483768	0.0041	0.0042	0.33
**rs732310**	T	G	0.52	85665	400902	0.95 (0.93-0.97)	1.33E-08	32.3	0.52	602539	-4.00E-04	0.0032	0.90
**rs7384878**	T	C	0.69	85934	401577	1.08 (1.06-1.1)	2.13E-18	75.8	0.68	483768	0.002	0.0044	0.64
**rs7421448**	T	C	0.097	85665	400902	0.9 (0.87-0.92)	1.01E-13	55.1	0.088	482750	0.0061	0.0072	0.40
**rs74504435**	A	G	0.91	85934	401577	1.09 (1.06-1.12)	2.05E-09	35.7	0.91	482750	-0.0106	0.007	0.13
**rs74685827**	T	G	0.98	85665	400902	0.82 (0.77-0.87)	8.63E-11	42.0	0.98	482750	-0.0047	0.0146	0.75
**rs74745468**	A	G	0.088	85934	401577	1.09 (1.06-1.12)	1.40E-08	32.1	0.084	483768	-0.018	0.0074	0.015
**rs7912495**	A	G	0.54	85934	401577	0.94 (0.93-0.96)	2.87E-12	48.7	0.55	483768	-0.0013	0.0041	0.75
**rs867230**	A	C	0.60	85934	401577	1.11 (1.09-1.12)	1.50E-33	144.3	0.59	482750	-0.0037	0.0042	0.37
**rs9676738**	A	G	0.042	85665	400902	1.18 (1.13-1.24)	1.24E-13	54.9	0.039	479952	-0.0081	0.0109	0.46
**rs976271**	A	G	0.36	85934	401577	1.05 (1.03-1.07)	3.24E-08	30.9	0.36	483768	0.0053	0.0043	0.21

Table e-9. Mendelian randomization estimates of the association between genetic susceptibility to AD with the level of physical activity.

Method	Association with AD	
	Estimate (SE)	P-value
Inverse variance weighted	-0.006 (0.008)	0.41
Weighted median	-0.006 (0.010)	0.52
MR Egger	0.004 (0.014)	0.80
Simple mode	-0.018 (0.018)	0.33
Weighted mode	-0.004 (0.013)	0.73
